# Antibacterial Activity of Bacteriocinogenic Commensal *Escherichia coli* against Zoonotic Strains Resistant and Sensitive to Antibiotics

**DOI:** 10.3390/antibiotics9070411

**Published:** 2020-07-15

**Authors:** Justyna Mazurek-Popczyk, Justyna Pisarska, Ewa Bok, Katarzyna Baldy-Chudzik

**Affiliations:** Department of Microbiology and Molecular Biology, Collegium Medicum, University of Zielona Góra, 65-417 Zielona Góra, Poland; j.pisarska@cm.uz.zgora.pl (J.P.); e.bok@cm.uz.zgora.pl (E.B.); k.baldy-chudzik@cm.uz.zgora.pl (K.B.-C.)

**Keywords:** *Escherichia coli*, bacteriocins, colicin, antibiotic resistance

## Abstract

Antibiotic resistance concerns various areas with high consumption of antibiotics, including husbandry. Resistant strains are transmitted to humans from livestock and agricultural products via the food chain and may pose a health risk. The commensal microbiota protects against the invasion of environmental strains by secretion of bacteriocins, among other mechanisms. The present study aims to characterize the bactericidal potential of bacteriocinogenic *Escherichia coli* from healthy humans against multidrug-resistant and antibiotic-sensitive strains from pigs and cattle. Bacteriocin production was tested by the double-layer plate method, and bacteriocin genes were identified by the PCR method. At least one bacteriocinogenic *E. coli* was detected in the fecal samples of 55% of tested individuals, adults and children. Among all isolates (*n* = 210), 37.1% were bacteriocinogenic and contained genes of colicin (Col) Ib, ColE1, microcin (Mcc) H47, ColIa, ColM, MccV, ColK, ColB, and single ColE2 and ColE7. Twenty-five *E. coli* carrying various sets of bacteriocin genes were further characterized and tested for their activity against zoonotic strains (*n* = 60). Strains with ColE7 (88%), ColE1-ColIa-ColK-MccH47 (85%), MccH47-MccV (85%), ColE1-ColIa-ColM (82%), ColE1 (75%), ColM (67%), and ColK (65%) were most active against zoonotic strains. Statistically significant differences in activity toward antibiotic-resistant strains were shown by commensal *E. coli* carrying MccV, ColK-MccV, and ColIb-ColK. The study demonstrates that bacteriocinogenic commensal *E. coli* exerts antagonistic activity against zoonotic strains and may constitute a defense line against multidrug-resistant strains.

## 1. Introduction

The intestinal microbiota is a highly diverse and dynamic community. It consists of indigenous flora and autochthonous microorganisms from the environment, which compete for niches and nutrients to colonize the intestinal habitat [1,2]. Protection of commensal species against invasion by exogenous microorganisms is a part of colonization resistance and involves the production of inhibitory molecules such as bacteriocins [3,4]. Bacteriocins are antimicrobial peptides, ribosomally produced with narrow killing spectra, produced by most bacteria [5]. 

*Escherichia coli* produces two classes of bacteriocins, colicins and microcins, which differ in their molecular weight, synthesis induction, mechanisms of secretion, and cytotoxicity [6]. Colicins are plasmid-encoded bacteriocins. Their operons, under the LexA promoter, include one to three genes responsible for the unique action of the system-toxin, immunity, and lysis. Colicin production is induced in stressful conditions that stimulate SOS responses or by nutrient depletion, but small amounts of colicins are also found to be produced spontaneously [7]. Concomitant synthesis of the immunity protein enables colicinogenic strains to be protected from the lethal effects of the protein toxin [8]. Lysis proteins, present in most colicin systems, allow the release of colicin into the environment but are also responsible for the death of the producer cell [7]. Colicins are proteins, >30 kDa, containing three functional domains [7,9]. They have been classified according to the translocation system to the target cells. Group A colicins use bacterial Tol-dependent translocation, whereas group B uses the Ton-system. Sensitive strains can be destroyed by various mechanisms. Pore-forming colicins depolarize the cytoplasmic membrane (ColA, ColE1, ColK, ColN, ColU, ColS4, ColB, ColIa, ColIb), enzymatic colicins have nuclease activity, e.g., DNase (ColE2, ColE7, ColE8, and ColE9), 16S rRNase (ColE3, ColE4, ColE6), and tRNase activity (ColE5, ColD), and ColM inhibits murein and O-antigen biosynthesis [7,9]. 

Microcins consist of a group of small (<5 kDa) plasmid-encoded peptides (MccB17, MccJ25) and larger (5–10 kDa) plasmid-mediated (MccL, MccV, and MccS) or chromosomally encoded microcins (MccM, MccH47). They are able to form pores in the bacterial membrane (MccV, MccL), inhibit the DNA gyrase GyrB (MccB17) and RNA polymerase and act on cytochromes (MccJ25), disrupt the cellular proton channel (MccH47 and probably MccM), and impair ATP synthase (MccH47) [10,11]. Microcins are not induced by the SOS system but are actively secreted upon nutrient depletion. Microcin-producing strains are immune to their own toxins, mostly by the production of immunity proteins and efflux pumps. Interaction with the target cells is through outer membrane receptors such as OmpF, OmpC, and receptors for iron uptake [11,12]. 

Sensitivity to bacteriocins depends on several factors, primarily the presence and availability of the receptor on the surface of the target cell and the presence of a functional system for bacteriocin translocation [7,13]. Cells can also be resistant, despite having receptors and an efficient transport system, due to the interaction of colicin with the immune protein when they themselves have genes encoding the given colicin [7]. 

Although *Escherichia coli* accounts for about 0.1% of the bacteria in the strict anaerobic environment of the human colon [14,15,16], it plays a significant role for the host organisms by producing vitamins and maintaining the anaerobic environment [17]. An important role in *E. coli* colonization resistance in the gastrointestinal tract involves the production of bacteriocins. The action of these inhibitory molecules, which affects phylogenetically related bacteria, can be vital for the health of the host organism. *E. coli* occurs not only as a commensal organism in the intestines of warm-blooded animals but also as six well-studied pathotypes causing morbidity and mortality [18,19,20,21,22]. What is more, *E. coli* bacteria in their primary intestinal environments are exposed to the antibiotics consumed by their host, so as a consequence, the high selection pressure results in the emergence of resistant strains [16]. Antibiotics are widely used in food production in farm animals, and therefore resistant and/or pathogenic strains may be transmitted to humans by the farm-to-table chain [23,24,25,26,27,28,29,30]. 

Studies on the activity of bacteriocins, as an alternative to adjunctive therapy with antibiotics, and trials focused on probiotic bacteria are intensively conducted [31,32,33,34]. They concern mostly the bacteriocins of Gram-positive bacteria [35,36,37,38,39], but colicins and microcins are also tested as proteins with activity against pathogenic Gram-negative bacteria [40,41,42,43,44,45,46,47,48,49,50], including a few studies related to antibiotic-resistant strains [51,52]. However, nonclinical strains of animal origin, derived from farm animals that are particularly resistant to antibiotics, may reveal a distinct profile of sensitivity to bacteriocins. The use of antibiotics during animal breeding creates specific conditions for the intensification of horizontal gene transfer events, creating a combination of numerous antibiotic resistance and bacteriocin genes carried on plasmids [24,33,53,54,55,56,57].

The aim of this study is to investigate the bactericidal potential of commensal, bacteriocinogenic *Escherichia coli* isolated from adults and children against multidrug-resistant strains of animal origin. The study constitutes a preliminary investigation of the activity of commensals against antibiotic-resistant strains. It is important to fully understand the ecological relationships between microorganisms inhabiting various niches in human organisms in order to understand the mechanisms leading to the expression and activity of bacteriocins in these environments, especially in the processes of combating pathogens and antibiotic-resistant strains.

## 2. Results

In total, from 100 fecal samples, 210 nonidentical *E. coli* bacteria were isolated (120 from adults, 90 from children). Phenotypic studies with the indicator strain revealed that 78 strains (37.1%) were bacteriocinogenic, including 42 strains from 28 adults and 36 strains from 27 children. 

The presence of one bacteriocin gene was detected in 32 strains, while co-occurrence of two or more colicin genes was found in 37 strains. In nine tested strains, none of the tested bacteriocin genes were detected. Among bacteriocinogenic strains, eight colicin genes-E1, E2, E7, Ib, Ia, M, B, K-and two microcin genes-H47 and V-were detected (Table 1). The colicin Ib gene (*n* = 23) and E1 (*n* = 22) were the most frequently detected, then microcin H47 (*n* = 17), colicin Ia (*n* = 16) and M (*n* = 15), microcin V (*n* = 11), colicin K (*n* = 10), B (*n* = 8), and single ColE2, ColE7. Colicins E1, Ib and Ia were detected more frequently in tested isolates from adults, while microcin H47 and colicin M were more frequently detected in *E. coli* from children.

Twenty-five strains (12 from adults and 13 from children) carrying various bacteriocins and bacteriocin sets (Table 2) were further characterized and tested for their activity against strains derived from animals.

Bacteriocinogenic strains showed varied bactericidal activity in relation to zoonotic *E. coli*, inhibiting the growth from 88% to 0% of tested strains (Table 2). In the group of strains carrying single colicin genes, the most frequent bactericidal activity was observed for the strain carrying the ColE7 gene (88.3%), then ColE1 (75%), ColM (66.7%), and ColK (65%). Strains with the most commonly identified colicin Ib gene (single or in the set with other bacteriocin genes) showed low bactericidal activity (from 0% to 41.6%), except the co-occurrence with microcin V (60%). Three *E. coli* carrying multiple bacteriocin genes for bacteriocins-MccH47-MccV (85%), ColE1-ColIa-ColK-MccH47 (85%), and ColE1-ColIa-ColM (81.6%)-revealed the highest antagonistic activity.

Most of the commensal *E. coli* showed similar activity against antibiotic-resistant and antibiotic-sensitive strains; however, there was a statistically significant difference in the bactericidal activity toward antibiotic-resistant strains for four *E. coli* with detected bacteriocins: MccV, ColK-MccV, ColIb-ColK, and ColE1-ColIa-ColB-ColM. These strains showed effective bactericidal activity from 18.3% to 43.3%.

Strains carrying varied sets of bacteriocin genes did not show clear synergistic effects. Commensal *E. coli* carrying two or three bacteriocins did not inhibit the growth of a larger number of zoonotic strains than *E. coli* with one bacteriocin. *E. coli* with four bacteriocins, despite a shared bacteriocin type, revealed totally different results (e.g., E1-Ib-K: 0%; E1-Ia-K-H47: 85%). 

**Figure 1:**
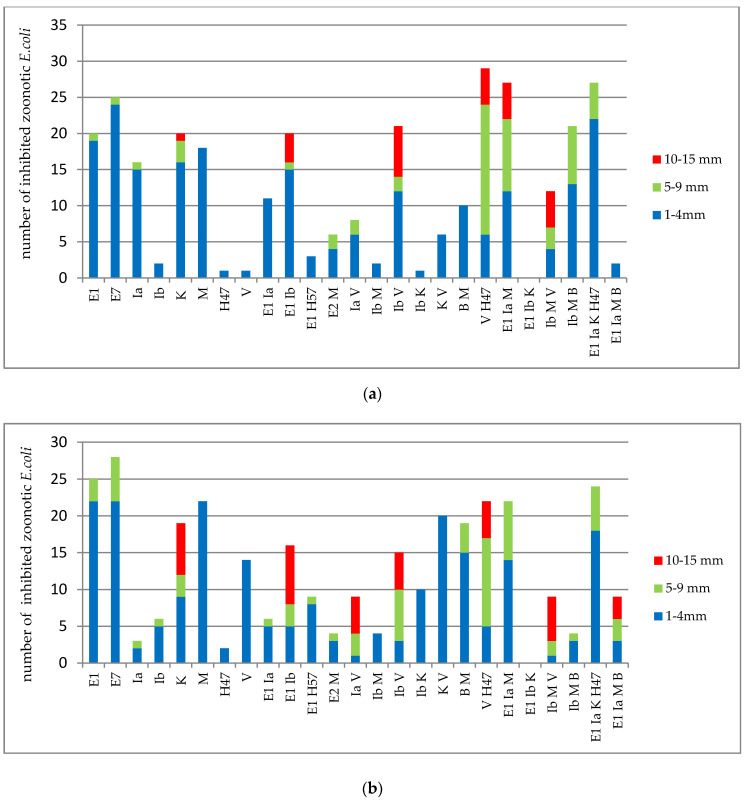
Differential bactericidal activity of bacteriocins produced by human *E. coli* strains expressed as the magnitude of the growth inhibition zone for antibiotic-sensitive (**a**) and -resistant (**b**) zoonotic *E. coli* strains.

Taking into account the bactericidal potency of bacteriocinogenic strains, based on the inhibition zone size, strains with single bacteriocins (E1, E7, Ia, Ib, M, MccV, MccH47) revealed low (1–4 mm) or medium (5–9 mm) antagonistic activity against zoonotic strains (Figure 1). The only exception was colicin K, where 13% of strains showed large (10–15 mm) inhibition zones. Strains with two and three bacteriocins more often revealed medium and large growth inhibition zones of zoonotic strains. No statistically significant difference in the bactericidal potency against antibiotic-sensitive and antibiotic-resistant strains was observed. 

## 3. Discussion

The scale of antibiotic resistance in the population is vast and much effort is put into its limitation [58]. High prevalence of antibiotic resistance among farm animals is the result of widespread and excessive use of antibiotics during animal husbandry [57,59,60]. Zoonotic strains transmitted to humans by food can induce a disease process or try to settle in the intestines of the host. *E. coli* is an important species transmitted from livestock and agricultural products [26,27,61,62,63]. An element of the natural protective barrier against infections or colonization by pathogenic and antibiotic-resistant bacteria is the commensal microbiota [39,51,64]. Production of bacteriocins plays an important role in the defense. These inhibitory molecules affect phylogenetically related bacteria and can be produced by commensals such as *E. coli* strains. In our study, bacteriocinogenic *E. coli* strains were detected in 55% of individuals of the tested healthy group. The frequency of bacteriocin production among fecal *E. coli* strains varied, but according to many researchers, it does not exceed this value and is most often around 50% [6,65,66,67]. Bacteriocin genes detected among *E. coli* from the study population encode quite homogeneous bacteriocins in terms of the mechanism of action concerning the cell membrane. Pore-forming colicins ColIb, ColE1, ColIa, ColK, ColB, microcin MccV, and cellular proton channel disruption MccH47 prevailed. ColM, inhibiting peptidoglycan synthesis, was most frequently detected in strains from children. The difference in the prevalence of bacteriocins between the groups of adults and children was also in regard to the presence of ColE2 and ColE7 DNA cleavage colicins. The structure of the microflora is variable over time and showed greater genetic diversity in adults [68], but as we can observe, the bacteriocin-producing strains, which promote competitive advantage, are present from an early age. 

The most common *E. coli* bacteriocins, detected in various human populations, are ColIa, ColE1, ColIb, ColM, ColB, and ColK [6,65,66,69,70,71]. Among microcins, MccH47 and MccV prevail [6,11,65,66,69,70,71], and microcin M is also frequently detected [6,11,65,69,70]. *E. coli* producing one type of bacteriocin is detected as often as multiple producers. Both the co-occurrence of bacteriocins that are similar to our research and those unique to the tested populations are observed. As Smajs suggested, the frequency of individual bacteriocin types can be geographically specific [66]. Differentiation in the detected bacteriocins increases among *E. coli* strains isolated from people with various medical conditions [41,70,71,72]; for example, colicins with nuclease activity are indicated to be attributed to the pathogenic strains [73].

The study revealed that commensal bacteriocinogenic *E. coli* strains have antagonistic activity against zoonotic strains. This activity is mainly caused by ColE7-, ColE1-, ColM-, and ColK-positive strains. The largest numbers of *E. coli* zoonotic strains were killed by DNA-degraded colicin ColE7, although without an advantage toward multi-resistant strains. The high sensitivity of strains from animals can be explained by the low prevalence of colicin producers in the populations. Sensitivity to the bacteriocins results from the presence of the appropriate receptors on the outer membrane, function of the bacteriocin transfer system, and finally, from the production of immune proteins when strains are able to produce specific bacteriocins themselves [7]. The availability of mechanisms enabling the introduction of colicin into the target cell in the absence of immune proteins results in high antagonistic activity in the population. ColE7 is a rare colicin among commensal flora. In our study, it was detected only in one strain (0.5% of all those tested); in literature data, it was found in 2.3% [6] and 1.7% [71] of fecal isolates. This colicin was also found in small amounts among *E. coli* causing asymptomatic bacteriuria [72], urinary tract infection [71], and in fecal samples from patients with different diseases [65], not exceeding 4%. Confirmation of the bactericidal effectiveness of this colicin was presented by Budič, where purified colicin inhibited the growth of 87% of tested *E. coli* strains from patients with bacteremia [41].

The most commonly identified in our tested set of strains, colicin Ib, showed low bactericidal activity (13.3%). It was similar when it occurred in sets with other bacteriocins, except co-occurrence with ColE1 (60%) and microcin V (60%). It can be assumed that the production of many bacteriocins that use different receptors, translocation systems, or mechanisms of action will allow a stronger bactericidal effect. Colicins Ib and ColE1 use different receptors (Cir receptor for catecholate siderophores and BtuB–cyanocobalamin transporter, respectively) and translocation systems (TonB–ExbBD and TolCAQ, respectively) [7], although they are pore-formation colicins. Their co-occurrence results in greater bactericidal activity, observed as larger diameters of the growth inhibition zone, but not as an increase in the number of destroyed zoonotic strains, compared to the activity of the monoproducers (Col E1-75%). Greater potency was also observed for the strain with the set of ColIb and MccV, despite these bacteriocins not only having a similar mechanism of action but also the same receptor (Cir protein receptor) and TonB transport pathway into the cell [73]. Another combination occurs in the strain carrying ColIb and ColM. They use different receptors (Cir and FhuA), but the same TonB transport systems and ColM inhibits cell wall synthesis. *E. coli* with ColIb and ColM acted against only 10% of zoonotic strains, whereas the monoproducers of ColIb and ColM were able to inhibit the growth of 13.3% and 66.6% of animal strains. It appears that colicin Ib does not have major antagonistic importance in the studied population.

*E. coli* strains carrying single genes of microcins MccH47 and MccV showed low antimicrobial activity (5% and 25%, respectively), while the strain carrying the genes of both of these microcins was able to inhibit 85% of the zoonotic strains. MccV, despite a low rate of growth inhibition, was mostly effective against multidrug-resistant strains, also in co-occurrence with ColK. Different indications regarding the activity of microcins against antibiotic-resistant strains were presented by Palmer et al. [52], who showed that purified MccH47 has broad and potent activity against medically relevant multidrug-resistant members of *Enterobacteriaceae.*

As can be observed, the bacteriocin activity detected in monoproducers did not correspond to their activity in the multiproducer. In many cases, no synergistic effect was noted. The results can be different when purified colicins are studied. Thus, for example, Budič observed that a colicin mix (E1, E6, E7, K, and M) acted better and was effective against 98% of *E. coli* from bacteremia [41]. It is likely that in the bacteriocin operons of naturally occurring strains, some mutation prevents the effective synthesis, secretion, or attack of the target cell. Another issue is the question of whether the strain carrying numerous bacteriocin genes produces all bacteriocins at once to launch a “massive attack” on the invader’s cell. Bacteriocin expression is triggered by stress conditions, but not all mechanisms are the same. Bacteriocin expression is a costly trait, so perhaps not all of them are expressed simultaneously. There are no studies showing whether all bacteriocins of a multiproducer are secreted into the environment. Bacteriocin activity studies are usually conducted on purified colicins or strains with operons of one bacteriocin, introduced by genetic engineering methods [41]. The abovementioned colicin M, occurring with ColIa and ColE1 (they differ in receptors and import system), shows 82% activity against zoonotic strains. Colicin M occurring with colicin Ib (closely related to ColIa) shows low activity (10%), although they also use different receptors and there was no competition for the binding site. Similarly, in the presented results, the strain with bacteriocins ColE1-ColIb-ColK (all pore-forming but with individual receptors) did not affect any of sixty tested animal strains (although it was able to inhibit the growth of the control strain), but when microcin MccH47 was “added” in the other strain, 85% of zoonotic strains were eliminated. Therefore, in the case of multibacteriocin producers, their antimicrobial effect appears to be the result of many factors affecting the sensitivity of the target cell.

The growth of multidrug-resistant zoonotic strains was inhibited by the bacteriocins with similar frequency as antibiotic-sensitive strains in most cases. Three strains inhibited the growth of antibiotic-resistant *E. coli* more frequently than antibiotic-sensitive *E. coli*, with a highly statistically significant difference: MccV, ColK-ColV, and ColIb-ColK. Taking into account the activity of *E. coli* with microcin V, the above sets of bacteriocins, and the activity of individual bacteriocins in the collection, one can identify two bacteriocins that are most active against multidrug-resistant bacteria: MccV and ColK. This indicates the potential of commensal *E. coli* strains to defend against the invasion of zoonotic, antibiotic-resistant strains, which should be further investigated. 

The antagonistic activity of a bacteriocinogenic strain in a complex population does not depend only on its activity but also on the susceptibility of other strains and the environment. As was already mentioned, the reason for the insensitivity of the cells to the bacteriocins may result from the lack of appropriate receptors or a disorder of the bacteriocin transfer system that makes the cells tolerant. In vivo, prevailing conditions may lead in an additional way to the strains’ antagonism. In the natural intestinal environment, other factors such as competition for the receptors of bacteriocin and the natural ligands may be relevant [7]. It has been shown that colicins compete with the corresponding natural ligand for the receptors. For example, cobalamin protects the BtuB receptor against E-type colicins or using ferrichrome blocks the FhuA receptor and action of colicin M [7]. According to the latest research, the density of the O-antigen also influences colicin sensitivity [13]. It has been shown that minor changes in growth conditions influence the density of LPS and hence sensitivity toward colicin. It was suggested that colicin could be more effective than is presented [13]. Colicin activity in vivo depends primarily on the colicin synthesis activated by the SOS system. In in-vitro studies, the SOS system is activated by nutrient depletion or the use of mitomycin, but other inducing factors are achievable in the natural intestinal environment. SOS can be triggered by antibiotics, which affect DNA replication such as quinolones or induce membrane stress such as b-lactams [74,75,76]. Additionally, oxidative stress induced by reactive oxygen [77] and some nutrient conditions appear prone to producing and releasing colicins [78]. The main argument indicating bacteriocin activity in the natural habitats are in vitro studies showing that bacteriocin-producing strains can enhance colonization resistance and protect against pathogens.

A practical approach to using direct competition of bacteriocinogenic commensals in therapy has been recently tested on Gram-positive enterococci by Kommineni [39]. The in vivo studies on mice have shown that bacteriocin expressed by commensals can influence niche competition in the gastrointestinal tract and eliminate intestinal colonization by multidrug-resistant bacteria. These studies indicate the possibility of a novel therapeutic strategy to eliminate antibiotic-resistant enterococci from the human gastrointestinal tract and, thereby, prevent the emergence of resistant enterococcal infections [39]. Another example is the study of Bosák et al., revealing an increase of colonization capacity by strains producing colicin F_Y_ against *Yersinia enterocolitica* infection [50].

The in vitro studies have shown the activity of bacteriocins produced by Gram-negative bacteria under culture conditions in agar [6,41,65,69,71] or liquid solution media [41,45,48,49], but in vivo [37] approaches can prove the effectiveness of bacteriocin antagonistic actions. Therefore, our research should be continued for a full understanding of the expression and activity of the bacteriocins, which play a role in the colonization resistance, protecting against multidrug-resistant strains of animal origin.

## 4. Materials and Methods 

### 4.1. Bacterial Strains

Commensal *E. coli* bacteria were isolated from healthy individuals, fifty adults (aged 18–56 years) and fifty children (aged from 6 months to 3 years) from Lubuskie Province in Poland. The people were from separate households, not related. Strains were isolated and identified from stool samples, as described earlier [68]. Briefly, samples were inoculated on mFC medium and incubated in 44 °C; next, characteristic blue colonies were transferred to MacConkey medium and identified in biochemical tests: Indole production, Methyl red reaction, Voges-Proskauer reaction, and Citrate utilization (IMVC). Five random *E. coli* strains were isolated from a single sample and then distinguished at the bases of BOX-PCR profiles [3,79]. As a result, 210 genetically diverse strains were selected for further studies. This study was approved by the bioethics committee at the District Medical Council in Zielona Góra (No. 03/85/2018).

*E. coli* bacteria from animals were isolated from fecal samples from pigs and cattle from two farms in Lubuskie Province in Poland. *E. coli* was isolated and identified using biochemical tests, as described above. One strain from one animal was further characterized. Antibiotic sensitivity was tested for ampicillin, cefotaxime, ceftazidime, tetracycline, streptomycin, gentamicin, nalidixic acid, ciprofloxacin, sulfamethoxazole, trimethoprim, and chloramphenicol, using the broth dilution method as described previously [60]. From over one hundred strains with different resistance profiles, thirty *E. coli* strains susceptible to antibiotics, and thirty multidrug-resistant strains were randomly selected for further research. 

### 4.2. Detection of Bacteriocins Producers among E. coli from Humans 

The tests were conducted using the double-layer method. The agar plates were inoculated by spots of 10 µL of overnight LB cultures of tested *E. coli* isolates from humans. Cultures were incubated at 37 °C for 48 hours. Bacteria were killed using chloroform vapors. Next, plates were overlaid with a soft agar containing 100 µL of an indicator strain *E. coli* K12-Row (PCM 827) that is sensitive to bacteriocins (OD_600_ approximately 0.05). The cultures were incubated at 37 °C overnight and examined for inhibition zones, which were measured in millimeters. In order to detect the activity of microcins H47 and M, which are sensitive to chloroform [65,80], tests were repeated without killing the bacteriocin producers. In such tests, the resulting growth inhibition zones are slightly less clear and sharp compared to the use of chloroform but sufficient for detection.

### 4.3. Identification of Bacteriocins Genes 

The presence of the bacteriocin genes was detected by the PCR method developed by Smajs using multiplex and uniplex reactions [71]. The presence of the colicin genes A, B, D, El, E2, E3, E6, E7, Ia/Ib, K, and M was detected, as well as microcins B17, H47, J25, M, and V. Thermal lysates constituted the DNA source. Strains for positive controls derived from the Polish Collection of Microorganisms, Wrocław, Poland (WFCC No. 106): *C. freundii* PCM 1632-ColA, *E. coli* K260-ColB, *E. coli* CA23-ColD, *E. coli* K53-ColEl, *E. coli* CA42-ColE2, *E. coli* M4 CA38-ColE3, *E. coli* CT14-ColE6; *E. coli* K317-ColE7; *E. coli* CA53-ColIa; *E. coli* MR2-ColIb; *E. coli* K49-ColK; *E. coli* 32T19/V75-ColM, *E. coli* 185Nx II9s/a-MccB17, *E.coli* CA58-MccH47, *E. coli* D55/1-MccJ25; *E.coli* CA46-MccM; *E. coli* PCM 1629-MccV.

### 4.4. Evaluation of Antibacterial Activity of Bacteriocinogenic E. coli against Zoonotic Strains 

The test was carried out as described above, with the difference that bacteriocin producers were grown as spots on the first agar layer and were overlaid with a second agar layer containing the zoonotic strain. 

### 4.5. Statistical Analysis

Pearson’s chi-squared test was used to assess the significance of differences in bactericidal activity against resistant and antibiotic-sensitive strains, with the significance level set at *p* < 0.05. The chi-square calculations were conducted for expected values equal to 5 or higher. The statistical analyses were performed using the program GraphPad (GraphPad Software Inc., San Diego, CA, USA). 

## 5. Conclusions

Antibiotic resistance is a serious health problem and arises from the high antibiotic consumption in different areas such as medicine, veterinary, and husbandry. Taking into account the epidemiological threat arising from the transmission of resistant strains of animal origin to humans, we undertook preliminary research on using natural interactions between microorganisms to tackle this problem. Colonization resistance involves the production of bacteriocins that destroy related strains, and we wanted to estimate the potential of commensal strains from healthy humans to defend against zoonotic strains. The study revealed that bacteriocinogenic commensal *E. coli* strains show antagonistic activity against strains of animal origin and some of them, particularly those carrying microcin V and colicin K, can be active against multidrug-resistant bacteria.

## Figures and Tables

**Table 1:** Prevalence of bacteriocin genes in the collection of bacteriocinogenic commensal *E. coli* isolates from healthy adults and children.

Bacteriocin Type	Number/Percent of Bacteriocin Genes Detected in *E. coli* From		Total Bacteriocinogenic (*n* = 78)
	Adults (*n* = 42)	Children (*n* = 36)	
E1	17/40.5%	5/13.9%	22/28.2%
E2	0	2/5.5%	2/2.5%
E7	1/2.4%	0	1/1.3%
Ia	11/26.2%	5/13.9%	16/20.5%
Ib	17/40.5%	6/16.6%	23/29.5%
K	9/21.4%	0	9/11.5%
M	3/7.1%	12/33.3%	15/19.2%
B	2/4.8%	6/16.7%	8/10.2%
MccH47	4/9.5%	13/36.1%	17/22.1%
MccV	5/11.9%	6/16.7%	11/14.1%

**Table 2:** Bactericidal potency of bacteriocinogenic commensal *E. coli* isolates from healthy adults and children, in relation to zoonotic strains resistant and sensitive to antibiotics.

Strains Carried Bacteriocin Genes	Number/Percent of Zoonotic Strains Inhibited by Bacteriocinogenic *E. coli*			*p*-Value
	Total (*n* = 60)	Resistant to Antibiotics (*n* = 30)	Sensitive to Antibiotics (*n* = 30)	
E1	45/75%	25/83.3%	20/66.7%	0.4561
E7	53/88.3%	28/93.3%	25/83.3%	0.6803
Ia	19/32%	3/10%	16/53.3%	0.0029
Ib	8/13.3%	6/20%	2/6.7%	-
K	39/65%	19/63.3%	20/66.7%	0.8728
M	40/66.6%	22/73.3%	18/60%	0.5271
MccH47	3/5%	2/6.7%	1/3.3%	-
MccV	15/25%	14/46.7%	1/3.3%	0.0008
E1, Ia	17/28.3%	6/20%	11/36.7%	0.2253
E1, Ib	36/60%	16/53.3%	20/66.7%	0.5050
E1, MccH47	12/20%	9/30%	3/10%	0.0833
E2, M	10/16.6%	4/13.3%	6/20%	-
Ia, MccV	17/28.3%	9/30%	8/26.7%	0.8084
Ib, M	6/10%	4/13.3%	2/6.7%	-
Ib, MccV	36/60%	15/50%	21/70%	0.3173
Ib, K	11/18.3%	10/33.3%	1/3.3%	0.0067
K, MccV	26/43.3%	20/66.7%	6/20%	0.0060
B, M	29/48.3%	19/63.3%	10/33.3%	0.0947
MccV, MccH47	51/85%	22/73.3%	29/96.7%	0.3270
E1, Ia, M	49/81.6%	22/73.3%	27/90%	0.4751
E1, Ib, K	0%	0%	0%	-
Ib, M, MccV	21/35%	9/30%	12/40%	0.5127
Ib, B, M	25/41.6%	4/13.3%	21/70%	0.0007
E1, Ia, K, MccH47	51/85%	24/80%	27/90%	0.6744
E1, Ia, B, M	11/18.3%	9/30%	2/6.7%	0.0348

Gray color—difference is considered to be statistically significant (*p* < 0.05) for bactericidal frequency against antibiotic-resistant zoonotic strains. Chi-square and *p*-value were not calculated for values below 10.
